# Bis(μ-4-phenyl­pyridine *N*-oxide-κ^2^ *O*:*O*)bis­[bis­(1,1,1,5,5,5-hexa­fluoro­pentane-2,4-dionato)copper(II)]

**DOI:** 10.1107/S1600536810022567

**Published:** 2010-06-23

**Authors:** Ana I. Ramos, José A. Fernandes, Patrícia Silva, Paulo J. A. Ribeiro-Claro, Susana S. Braga, Filipe A. Almeida Paz

**Affiliations:** a Department of Chemistry, CICECO, University of Aveiro, 3810-193 Aveiro, Portugal

## Abstract

The asymmetric unit of the title compound, [Cu_2_(C_5_HF_6_O_2_)_4_(C_11_H_9_NO)_2_], contains one half of the centrosymmetric dinuclear complex. The coordination geometry of the Cu^II^ atom is octa­hedral, exhibiting a typical Jahn–Teller distortion. One trifluoro­methyl group is rotationally disordered between two orientations in a 1:1 ratio.

## Related literature

For the use of copper complexes in optical devices, see: Akkılıç *et al.* (2010 ▶); Armaroli *et al.* (2007 ▶); Bessho *et al.* (2008 ▶); Chan *et al.* (2010 ▶); Daniel *et al.* (2009 ▶); Jeon *et al.* (2008 ▶); Kambayashi *et al.* (2005 ▶); Peranantham *et al.* (2007 ▶); Si *et al.* (2008 ▶); Vogler & Kunkely (2001 ▶); Walsh *et al.* (2009 ▶). For related complexes with 4-phenyl­pyridine-*N*-oxide, see: Papadaki *et al.* (1999 ▶); Watson & Johnson (1971 ▶). For general background to studies on copper complexes from our research group, see: Fernandes *et al.* (2010 ▶); Shi *et al.* (2006 ▶); Paz *et al.* (2005 ▶); Girginova *et al.* (2005 ▶); Brandão *et al.* (2005 ▶). For a description of the Cambridge Structural Database, see: Allen (2002 ▶).
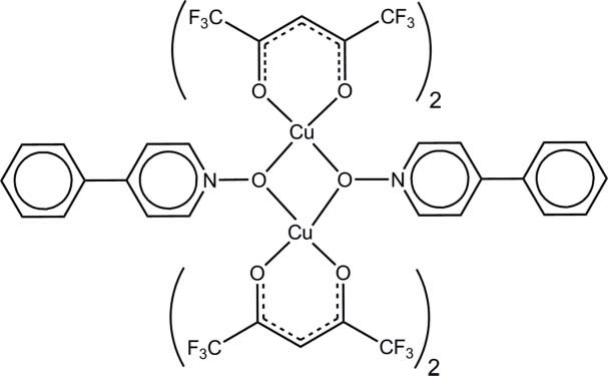


## Experimental

### 

#### Crystal data

[Cu_2_(C_5_HF_6_O_2_)_4_(C_11_H_9_NO)_2_]
*M* *_r_* = 1297.70
Triclinic, 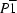

*a* = 10.3830 (4) Å
*b* = 10.7870 (4) Å
*c* = 11.2498 (4) Å
α = 99.055 (2)°
β = 99.036 (2)°
γ = 98.258 (2)°
*V* = 1210.40 (8) Å^3^
*Z* = 1
Mo *K*α radiation
μ = 1.03 mm^−1^
*T* = 150 K
0.18 × 0.10 × 0.09 mm

#### Data collection

Bruker X8 Kappa CCD APEXII diffractometer
Absorption correction: multi-scan (*SADABS*; Sheldrick, 1997 ▶) *T*_min_ = 0.836, *T*_max_ = 0.913
47759 measured reflections
6427 independent reflections
5461 reflections with *I* > 2σ(*I*)
*R*_int_ = 0.029

#### Refinement

*R*[*F* ^2^ > 2σ(*F* ^2^)] = 0.048
*wR*(*F* ^2^) = 0.139
*S* = 1.04
6427 reflections
397 parameters
24 restraints
H-atom parameters constrained
Δρ_max_ = 1.21 e Å^−3^
Δρ_min_ = −1.40 e Å^−3^

### 

Data collection: *APEX2* (Bruker, 2006 ▶); cell refinement: *SAINT-Plus* (Bruker, 2005 ▶); data reduction: *SAINT-Plus*; program(s) used to solve structure: *SHELXTL* (Sheldrick, 2008 ▶); program(s) used to refine structure: *SHELXTL*; molecular graphics: *DIAMOND* (Brandenburg, 2009 ▶); software used to prepare material for publication: *SHELXTL*.

## Supplementary Material

Crystal structure: contains datablocks global, I. DOI: 10.1107/S1600536810022567/cv2732sup1.cif

Structure factors: contains datablocks I. DOI: 10.1107/S1600536810022567/cv2732Isup2.hkl

Additional supplementary materials:  crystallographic information; 3D view; checkCIF report

## Figures and Tables

**Fig. 1.**
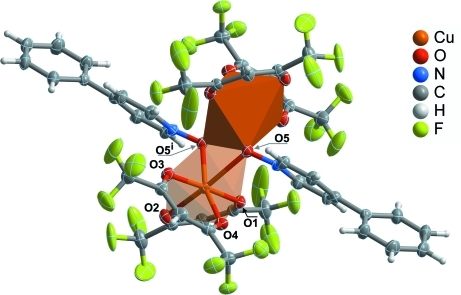
Schematic representation, in ball-and-stick and polyhedral fashions, of the molecular structure of the title compound. The crystallographic disorder associated with the —CF3 group was omitted for simplicity. Symmetry code: (i) 2 - x, -y, -z.

**Fig. 2.**
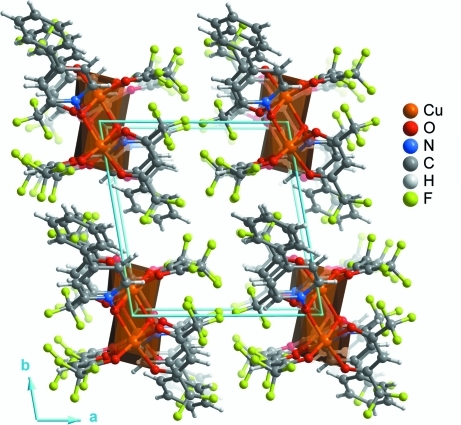
Perspective view along the c axis of the unit cell of the crystal packing of the title compound.

## supplementary crystallographic information

### Comment

The coordination chemistry of 4-phenylpyridine-*N*-oxide (PPNO; C_11_H_9_NO) is rather unknown. Surveying the Cambridge Structural Database (Allen, 2002) only two coordination compounds were found, namely a copper (Watson & Johnson, 1971) and a tin compound (Papadaki *et al.*, 1999).

The title compound, [Cu(C_5_HF_6_O_2_)_2_(C_11_H_9_NO)_2_] (I) (where C_5_HF_6_O_2_^-^ is hexafluoroacetylacetonate and C_11_H_9_NO corresponds to 4-phenylpyridine-*N*-oxide), has a bright green colour which is of interest for the potential use as a dye in several applications, such as in organic electronics. Coloured materials, including metal coordination compounds, have been explored in the last decades because pigmentation is a strong indicative of promising physical and optical properties (Vogler & Kunkely, 2001). Recent applications of copper coordination complexes include a Schottky diode based on a macrocyclic binuclear Cu^2+^ complex (Akkılıç *et al.*, 2010) and a series of Graetzel solar cells using 6,6'-dimethyl-dicarboxylated-bipyridine complexes (Bessho *et al.*, 2008). The use of Cu^2+^ complexes in OLEDs is still limited (Armaroli *et al.*, 2007), and emission still relies mostly on Cu^+^ complexes, such as [Cu(I)*bis*(triphenylphosphine)dipyridophenazine] (Walsh *et al.*, 2009) and three Cu(I)pyridylbenzimidazole complexes (Si *et al.*, 2008). In addition, copper complexes are advantageous from the technological processing perspective, adaptable to various film deposition techniques: molecular beam epitaxy (Kambayashi *et al.*, 2005), vacuum evaporation (Peranantham *et al.*, 2007), electrodeposition (Chan *et al.*, 2010) and chemical vapour deposition (CVD), either plasma-enhanced (Daniel *et al.*, 2009) or the widespread metal organic CVD (Jeon *et al.*, 2008).

Following our interest in the preparation and study of the properties of copper compounds (Fernandes *et al.*, 2010; Shi *et al.*, 2006; Paz *et al.*, 2005; Girginova *et al.*, 2005; Brandão *et al.*, 2005) we have reacted [Cu(hfac)_2_] (where hfac^-^ stands for hexafluoroacetylacetonate) with PPNO, affording the title compound as a vivid green crystalline material, whose crystal structure we report herein.

The structure is dimeric having a point of inversion in the geometrical centre of the quadrangle defined by Cu1···O5···Cu1^(i)^···O5^(i)^ (Figure 1) [symmetry code: (i) 2 - *x*, -*y*, -*z*]. The asymmetric unit contains one PPNO ligand and one [Cu(hfac)_2_] moiety. The atoms Cu1, O2, O4 and O5, and their symmetry-related counterparts [symmetry code (i)], form a plane (longest distance to the average plane of about 0.041 Å). Likewise, atoms Cu1, O1 and O3, and their symmetry-related counterparts [symmetry code (i)], define a second plane (longest distance to this average plane of about 0.048 Å), which is perpendicular to the previous one. The Cu^2+^ centre exhibits a typical octahedral coordination environment with a strong Jahn-Teller distortion: the Cu—O bonds (equatorial plane) range from 1.9500 (19) to 1.9751 (18) Å, while the Cu—O bonds (at apical positions) are either 2.251 (2) or 2.4427 (18) Å. With the exception of the angles O5—Cu1—O5^(i)^ [76.90 (7)°] and O2—Cu1—O4 [101.08 (8)°], the *cis* octahedral angles fall within a rather short range around the ideal value: 84.55 (7)–97.37 (8)° (Table 1). The *trans* octahedral angles are between 161.06 (8)° and 174.96 (8)°. The two average planes containing the aromatic rings of PPNO are mutually rotated by *ca* 37°. The structure exhibits crystallographic disorder associated with the fluorine atoms F4, F5 and F6 (rates of occupancy 50% for each location). Noteworthy, symmetry-related copper complexes are located at the vertex of the unit cell (Figure 2), with the inner space being essentially occupied by the coordinated organic ligands, leading to a hydrophobic empty space of about 36 Å^3^.

### Experimental

Chemicals were purchased from commercial sources and were used as received without purification.

4-Phenylpyridine-*N*-oxide (PPNO, 71.7 mg, 0.419 mmol, Aldrich) was slowly added to a previously-prepared [Cu(hfac)_2_] (100.0 mg, 0.209 mmol; hfac^-^ stands for hexafluoroacetylacetonate) solution in 10 ml of acetone. The resulting solution was magnetically stirred at 30 °C for 30 minutes. By slow evaporation to dryness, a green solid was obtained, which was dissolved in ethanol, gravity filtered and deposited above a water layer. Green crystals of the title compound formed after two days.

### Refinement

Hydrogen atoms bound to carbon were located at their idealized positions and were included in the final structural model in riding-motion approximation with C—H = 0.95 Å. The isotropic displacement parameters for these atoms were fixed at 1.2 times *U*_eq_ of the respective carbon atom.

The substituent —CF_3_ groups were found to be severely affected by thermal disorder. Attempts to model this disorder were only successful for one moiety which was included in the final structure with two crystallographic positions (fixed rate of occupancy of 50% for each). In order to ensure a chemically reasonable geometry for this disorder moiety all C—F and F···F distances in the structure were restrained to common refineable values.

### Crystal data

**Table:**

[Cu_2_(C_5_HF_6_O_2_)_4_(C_11_H_9_NO)_2_]	*Z* = 1
*M**_r_* = 1297.70	*F*(000) = 642
Triclinic, *P*1	*D*_x_ = 1.780 Mg m^−^^3^
Hall symbol: -P 1	Mo *K*α radiation, λ = 0.71073 Å
*a* = 10.3830 (4) Å	Cell parameters from 9842 reflections
*b* = 10.7870 (4) Å	θ = 2.9–28.6°
*c* = 11.2498 (4) Å	µ = 1.03 mm^−^^1^
α = 99.055 (2)°	*T* = 150 K
β = 99.036 (2)°	Block, green
γ = 98.258 (2)°	0.18 × 0.10 × 0.09 mm
*V* = 1210.40 (8) Å^3^	

### Data collection

**Table:**

Bruker X8 Kappa CCD APEXII diffractometer	6427 independent reflections
Radiation source: fine-focus sealed tube	5461 reflections with *I* > 2σ(*I*)
graphite	*R*_int_ = 0.029
ω/phi scans	θ_max_ = 29.1°, θ_min_ = 3.7°
Absorption correction: multi-scan (*SADABS*; Sheldrick, 1997)	*h* = −14→14
*T*_min_ = 0.836, *T*_max_ = 0.913	*k* = −14→14
47759 measured reflections	*l* = −15→15

### Refinement

**Table:**

Refinement on *F*^2^	Primary atom site location: structure-invariant direct methods
Least-squares matrix: full	Secondary atom site location: difference Fourier map
*R*[*F*^2^ > 2σ(*F*^2^)] = 0.048	Hydrogen site location: inferred from neighbouring sites
*wR*(*F*^2^) = 0.139	H-atom parameters constrained
*S* = 1.04	*w* = 1/[σ^2^(*F*_o_^2^) + (0.0712*P*)^2^ + 2.0571*P*] where *P* = (*F*_o_^2^ + 2*F*_c_^2^)/3
6427 reflections	(Δ/σ)_max_ = 0.003
397 parameters	Δρ_max_ = 1.21 e Å^−^^3^
24 restraints	Δρ_min_ = −1.40 e Å^−^^3^

### Special details

**Table:**

Geometry. All e.s.d.'s (except the e.s.d. in the dihedral angle between two l.s. planes) are estimated using the full covariance matrix. The cell e.s.d.'s are taken into account individually in the estimation of e.s.d.'s in distances, angles and torsion angles; correlations between e.s.d.'s in cell parameters are only used when they are defined by crystal symmetry. An approximate (isotropic) treatment of cell e.s.d.'s is used for estimating e.s.d.'s involving l.s. planes.
Refinement. Refinement of *F*^2^ against ALL reflections. The weighted *R*-factor *wR* and goodness of fit *S* are based on *F*^2^, conventional *R*-factors *R* are based on *F*, with *F* set to zero for negative *F*^2^. The threshold expression of *F*^2^ > σ(*F*^2^) is used only for calculating *R*-factors(gt) *etc*. and is not relevant to the choice of reflections for refinement. *R*-factors based on *F*^2^ are statistically about twice as large as those based on *F*, and *R*- factors based on ALL data will be even larger.

### Fractional atomic coordinates and isotropic or equivalent isotropic displacement parameters (Å^2^)

**Table:**

	*x*	*y*	*z*	*U*_iso_*/*U*_eq_	Occ. (<1)
Cu1	1.01536 (3)	0.13209 (3)	−0.07044 (3)	0.02205 (10)	
O1	1.17596 (17)	0.20733 (17)	0.05247 (16)	0.0235 (4)	
O2	1.12021 (19)	0.2109 (2)	−0.21003 (18)	0.0312 (4)	
O3	0.86603 (19)	0.05900 (18)	−0.20302 (18)	0.0284 (4)	
O4	0.93026 (17)	0.27435 (17)	−0.01281 (18)	0.0255 (4)	
O5	0.91578 (18)	0.02993 (17)	0.08021 (16)	0.0238 (4)	
N1	0.8997 (2)	0.0995 (2)	0.18579 (19)	0.0217 (4)	
C1	1.4020 (2)	0.2518 (2)	0.14122 (18)	0.0382 (7)	
C2	1.2903 (2)	0.2335 (2)	0.0291 (2)	0.0227 (5)	
C3	1.3311 (3)	0.2502 (3)	−0.0794 (2)	0.0273 (5)	
H3	1.4233	0.2686	−0.0795	0.033*	
C4	1.2419 (3)	0.2411 (3)	−0.1900 (2)	0.0261 (5)	
C5	1.3046 (2)	0.2749 (3)	−0.2985 (2)	0.0426 (7)	
C6	0.6641 (2)	0.03964 (18)	−0.3416 (2)	0.0404 (7)	
C7	0.7645 (3)	0.1076 (3)	−0.2257 (2)	0.0272 (5)	
C8	0.7299 (3)	0.2135 (3)	−0.1602 (3)	0.0329 (6)	
H8	0.6459	0.2359	−0.1852	0.039*	
C9	0.8158 (2)	0.2876 (2)	−0.0585 (2)	0.0247 (5)	
C10	0.7717 (3)	0.4038 (3)	0.0107 (3)	0.0303 (6)	
C11	1.0036 (3)	0.1754 (3)	0.2619 (2)	0.0272 (5)	
H11	1.0903	0.1761	0.2446	0.033*	
C12	0.9841 (3)	0.2522 (3)	0.3650 (2)	0.0261 (5)	
H12	1.0580	0.3048	0.4192	0.031*	
C13	0.8573 (2)	0.2537 (2)	0.3912 (2)	0.0214 (4)	
C14	0.7527 (2)	0.1713 (3)	0.3101 (2)	0.0256 (5)	
H14	0.6651	0.1677	0.3258	0.031*	
C15	0.7754 (2)	0.0959 (2)	0.2086 (2)	0.0249 (5)	
H15	0.7035	0.0408	0.1539	0.030*	
C16	0.8344 (2)	0.3403 (2)	0.4986 (2)	0.0228 (5)	
C17	0.9250 (3)	0.3653 (3)	0.6087 (2)	0.0299 (5)	
H17	1.0018	0.3265	0.6148	0.036*	
C18	0.9035 (3)	0.4465 (3)	0.7091 (3)	0.0356 (6)	
H18	0.9657	0.4631	0.7837	0.043*	
C19	0.7924 (3)	0.5035 (3)	0.7018 (3)	0.0365 (6)	
H19	0.7783	0.5591	0.7711	0.044*	
C20	0.7019 (3)	0.4793 (3)	0.5936 (3)	0.0398 (7)	
H20	0.6254	0.5185	0.5884	0.048*	
C21	0.7225 (3)	0.3976 (3)	0.4916 (3)	0.0325 (6)	
H21	0.6598	0.3811	0.4173	0.039*	
F1	1.36023 (19)	0.2437 (2)	0.24323 (16)	0.0546 (6)	
F2	1.4801 (3)	0.3614 (3)	0.1595 (2)	0.1153 (16)	
F3	1.4749 (3)	0.1648 (3)	0.1247 (2)	0.150 (2)	
F4	1.3541 (8)	0.3951 (3)	−0.2833 (5)	0.122 (4)	0.50
F5	1.4012 (5)	0.2125 (7)	−0.3118 (5)	0.084 (3)	0.50
F6	1.2224 (4)	0.2467 (7)	−0.4024 (3)	0.0588 (17)	0.50
F4'	1.2595 (7)	0.3725 (7)	−0.3328 (9)	0.109 (4)	0.50
F5'	1.4329 (2)	0.3025 (8)	−0.2815 (7)	0.098 (4)	0.50
F6'	1.2680 (10)	0.1810 (7)	−0.3909 (6)	0.199 (8)	0.50
F7	0.7002 (2)	−0.0620 (2)	−0.3950 (2)	0.0803 (9)	
F8	0.6504 (3)	0.1140 (2)	−0.4212 (2)	0.0945 (12)	
F9	0.54684 (19)	0.0040 (2)	−0.3187 (2)	0.0723 (8)	
F10	0.85443 (17)	0.51032 (16)	0.01099 (18)	0.0366 (4)	
F11	0.7719 (2)	0.3931 (2)	0.12708 (19)	0.0483 (5)	
F12	0.65074 (17)	0.41987 (19)	−0.0375 (2)	0.0465 (5)	

### Atomic displacement parameters (Å^2^)

**Table:**

	*U*^11^	*U*^22^	*U*^33^	*U*^12^	*U*^13^	*U*^23^
Cu1	0.01770 (15)	0.02152 (16)	0.02440 (17)	0.00386 (11)	−0.00051 (11)	0.00057 (11)
O1	0.0194 (8)	0.0263 (9)	0.0232 (8)	0.0029 (7)	0.0011 (6)	0.0031 (7)
O2	0.0260 (9)	0.0402 (11)	0.0265 (9)	0.0039 (8)	−0.0006 (7)	0.0109 (8)
O3	0.0249 (9)	0.0284 (9)	0.0276 (9)	0.0032 (7)	−0.0020 (7)	0.0005 (7)
O4	0.0194 (8)	0.0235 (8)	0.0314 (9)	0.0055 (7)	−0.0009 (7)	0.0023 (7)
O5	0.0282 (9)	0.0230 (8)	0.0208 (8)	0.0081 (7)	0.0062 (7)	0.0007 (6)
N1	0.0212 (9)	0.0218 (9)	0.0222 (10)	0.0047 (8)	0.0049 (8)	0.0026 (8)
C1	0.0215 (12)	0.060 (2)	0.0305 (14)	0.0032 (13)	−0.0010 (11)	0.0098 (13)
C2	0.0196 (10)	0.0218 (11)	0.0250 (12)	0.0052 (9)	−0.0009 (9)	0.0031 (9)
C3	0.0187 (11)	0.0348 (14)	0.0291 (13)	0.0053 (10)	0.0032 (9)	0.0088 (10)
C4	0.0272 (12)	0.0279 (12)	0.0260 (12)	0.0100 (10)	0.0061 (10)	0.0074 (10)
C5	0.0358 (16)	0.065 (2)	0.0313 (15)	0.0122 (15)	0.0082 (12)	0.0154 (15)
C6	0.0387 (16)	0.0369 (16)	0.0368 (16)	−0.0026 (13)	−0.0103 (13)	0.0061 (13)
C7	0.0217 (11)	0.0285 (12)	0.0276 (13)	−0.0030 (10)	−0.0033 (9)	0.0077 (10)
C8	0.0198 (12)	0.0331 (14)	0.0421 (16)	0.0046 (10)	−0.0038 (11)	0.0054 (12)
C9	0.0197 (11)	0.0221 (11)	0.0330 (13)	0.0031 (9)	0.0046 (9)	0.0081 (10)
C10	0.0209 (12)	0.0301 (13)	0.0405 (15)	0.0068 (10)	0.0048 (10)	0.0062 (11)
C11	0.0189 (11)	0.0329 (13)	0.0283 (13)	0.0016 (10)	0.0064 (9)	0.0013 (10)
C12	0.0217 (11)	0.0303 (13)	0.0236 (12)	−0.0002 (10)	0.0041 (9)	0.0008 (10)
C13	0.0215 (11)	0.0227 (11)	0.0208 (11)	0.0045 (9)	0.0043 (9)	0.0049 (9)
C14	0.0180 (11)	0.0312 (13)	0.0268 (12)	0.0053 (9)	0.0039 (9)	0.0022 (10)
C15	0.0185 (11)	0.0270 (12)	0.0267 (12)	0.0034 (9)	0.0011 (9)	0.0008 (10)
C16	0.0240 (11)	0.0237 (11)	0.0202 (11)	0.0025 (9)	0.0047 (9)	0.0036 (9)
C17	0.0266 (13)	0.0378 (14)	0.0242 (12)	0.0063 (11)	0.0033 (10)	0.0033 (11)
C18	0.0371 (15)	0.0429 (16)	0.0214 (13)	0.0002 (13)	0.0017 (11)	−0.0002 (11)
C19	0.0498 (18)	0.0337 (15)	0.0253 (13)	0.0071 (13)	0.0126 (12)	−0.0019 (11)
C20	0.0433 (17)	0.0414 (17)	0.0357 (16)	0.0191 (14)	0.0085 (13)	−0.0012 (13)
C21	0.0330 (14)	0.0362 (14)	0.0270 (13)	0.0135 (12)	0.0007 (11)	0.0003 (11)
F1	0.0415 (11)	0.0933 (18)	0.0272 (9)	0.0156 (11)	−0.0025 (8)	0.0110 (10)
F2	0.084 (2)	0.159 (3)	0.0636 (18)	−0.081 (2)	−0.0360 (15)	0.046 (2)
F3	0.147 (3)	0.261 (5)	0.0483 (15)	0.177 (4)	−0.0346 (18)	−0.029 (2)
F4	0.228 (11)	0.058 (4)	0.073 (5)	−0.050 (5)	0.081 (7)	0.007 (3)
F5	0.048 (3)	0.180 (9)	0.061 (4)	0.065 (4)	0.038 (3)	0.063 (5)
F6	0.035 (2)	0.128 (6)	0.0152 (19)	0.018 (3)	0.0034 (15)	0.016 (3)
F4'	0.070 (4)	0.180 (10)	0.137 (8)	0.059 (5)	0.055 (5)	0.135 (8)
F5'	0.029 (2)	0.201 (10)	0.090 (5)	0.011 (4)	0.018 (3)	0.101 (7)
F6'	0.313 (19)	0.144 (9)	0.111 (8)	−0.082 (10)	0.155 (11)	−0.072 (7)
F7	0.0761 (18)	0.0833 (19)	0.0559 (15)	0.0243 (15)	−0.0267 (13)	−0.0345 (14)
F8	0.128 (3)	0.0745 (18)	0.0489 (14)	−0.0453 (18)	−0.0450 (15)	0.0346 (13)
F9	0.0367 (12)	0.0837 (18)	0.0747 (17)	−0.0197 (12)	−0.0092 (11)	−0.0045 (14)
F10	0.0320 (9)	0.0236 (8)	0.0516 (11)	0.0037 (7)	0.0063 (8)	0.0015 (7)
F11	0.0546 (12)	0.0560 (12)	0.0441 (11)	0.0220 (10)	0.0241 (10)	0.0124 (9)
F12	0.0235 (8)	0.0407 (10)	0.0724 (14)	0.0138 (7)	0.0003 (9)	0.0024 (9)

### Geometric parameters (Å, °)

**Table:**

Cu1—O1	1.9711 (17)	C7—C8	1.383 (4)
Cu1—O2	2.251 (2)	C8—C9	1.390 (4)
Cu1—O3	1.9500 (19)	C8—H8	0.9500
Cu1—O4	1.9524 (18)	C9—C10	1.535 (4)
Cu1—O5	2.4427 (18)	C10—F10	1.331 (3)
Cu1—O5^i^	1.9751 (18)	C10—F11	1.332 (4)
O1—C2	1.258 (3)	C10—F12	1.334 (3)
O2—C4	1.234 (3)	C11—C12	1.375 (4)
O3—C7	1.252 (3)	C11—H11	0.9500
O4—C9	1.256 (3)	C12—C13	1.395 (3)
O5—N1	1.349 (3)	C12—H12	0.9500
O5—Cu1^i^	1.9751 (18)	C13—C14	1.399 (4)
N1—C11	1.343 (3)	C13—C16	1.479 (3)
N1—C15	1.351 (3)	C14—C15	1.367 (4)
N1—Cu1^i^	2.945 (2)	C14—H14	0.9500
C1—F3	1.297 (4)	C15—H15	0.9500
C1—F1	1.300 (3)	C16—C21	1.390 (4)
C1—F2	1.301 (4)	C16—C17	1.396 (4)
C1—C2	1.540 (3)	C17—C18	1.384 (4)
C2—C3	1.383 (4)	C17—H17	0.9500
C3—C4	1.410 (4)	C18—C19	1.381 (5)
C3—H3	0.9500	C18—H18	0.9500
C4—C5	1.540 (3)	C19—C20	1.380 (5)
C5—F6'	1.2982 (15)	C19—H19	0.9500
C5—F4	1.2985 (15)	C20—C21	1.397 (4)
C5—F6	1.2987 (15)	C20—H20	0.9500
C5—F5'	1.2989 (15)	C21—H21	0.9500
C5—F4'	1.2998 (15)	F4—F4'	1.025 (7)
C5—F5	1.3003 (15)	F4—F5'	1.379 (6)
C6—F7	1.298 (3)	F5—F5'	0.968 (7)
C6—F8	1.298 (3)	F5—F6'	1.485 (7)
C6—F9	1.301 (3)	F6—F6'	0.921 (9)
C6—C7	1.542 (3)	F6—F4'	1.423 (7)
			
O1—Cu1—O2	87.50 (7)	O3—C7—C6	115.7 (2)
O1—Cu1—O5	91.03 (7)	C8—C7—C6	115.7 (2)
O1—Cu1—O5^i^	86.68 (8)	C8—C7—Cu1	94.49 (17)
O2—Cu1—O5	174.17 (7)	C6—C7—Cu1	149.74 (18)
O3—Cu1—O1	174.96 (8)	C7—C8—C9	121.1 (2)
O3—Cu1—O2	87.73 (8)	C7—C8—Cu1	60.54 (14)
O3—Cu1—O4	92.62 (8)	C9—C8—Cu1	60.60 (15)
O3—Cu1—O5	93.57 (7)	C7—C8—H8	119.4
O3—Cu1—O5^i^	92.35 (8)	C9—C8—H8	119.4
O4—Cu1—O1	89.86 (8)	Cu1—C8—H8	178.7
O4—Cu1—O2	101.08 (8)	O4—C9—C8	128.7 (3)
O4—Cu1—O5	84.55 (7)	O4—C9—C10	112.6 (2)
O4—Cu1—O5^i^	161.06 (8)	C8—C9—C10	118.7 (2)
O5^i^—Cu1—O2	97.37 (8)	C8—C9—Cu1	94.28 (17)
O5^i^—Cu1—O5	76.90 (7)	C10—C9—Cu1	146.95 (18)
C2—O1—Cu1	125.00 (16)	F10—C10—F11	107.2 (2)
C4—O2—Cu1	120.66 (17)	F10—C10—F12	107.2 (2)
C7—O3—Cu1	124.47 (18)	F11—C10—F12	107.7 (2)
C9—O4—Cu1	124.12 (18)	F10—C10—C9	110.8 (2)
N1—O5—Cu1^i^	123.68 (14)	F11—C10—C9	110.5 (2)
N1—O5—Cu1	120.78 (14)	F12—C10—C9	113.3 (2)
Cu1^i^—O5—Cu1	103.10 (7)	N1—C11—C12	119.8 (2)
C11—N1—O5	120.6 (2)	N1—C11—H11	120.1
C11—N1—C15	121.3 (2)	C12—C11—H11	120.1
O5—N1—C15	117.9 (2)	C11—C12—C13	120.9 (2)
C11—N1—Cu1^i^	109.45 (16)	C11—C12—H12	119.5
C15—N1—Cu1^i^	120.81 (16)	C13—C12—H12	119.5
F3—C1—F1	106.88 (13)	C12—C13—C14	117.0 (2)
F3—C1—F2	106.82 (13)	C12—C13—C16	121.4 (2)
F1—C1—F2	106.45 (13)	C14—C13—C16	121.6 (2)
F3—C1—C2	109.60 (18)	C15—C14—C13	120.6 (2)
F1—C1—C2	113.95 (18)	C15—C14—H14	119.7
F2—C1—C2	112.73 (19)	C13—C14—H14	119.7
O1—C2—C3	130.7 (2)	N1—C15—C14	120.2 (2)
O1—C2—C1	113.9 (2)	N1—C15—H15	119.9
C3—C2—C1	115.4 (2)	C14—C15—H15	119.9
C3—C2—Cu1	97.93 (16)	C21—C16—C17	119.1 (2)
C1—C2—Cu1	145.40 (16)	C21—C16—C13	120.6 (2)
C2—C3—C4	122.9 (2)	C17—C16—C13	120.3 (2)
C2—C3—H3	118.5	C18—C17—C16	120.3 (3)
C4—C3—H3	118.5	C18—C17—H17	119.9
O2—C4—C3	128.2 (2)	C16—C17—H17	119.9
O2—C4—C5	116.2 (2)	C19—C18—C17	120.6 (3)
C3—C4—C5	115.6 (2)	C19—C18—H18	119.7
C3—C4—Cu1	89.46 (16)	C17—C18—H18	119.7
C5—C4—Cu1	154.41 (17)	C20—C19—C18	119.7 (3)
F6'—C5—F4	135.8 (5)	C20—C19—H19	120.1
F4—C5—F6	106.84 (14)	C18—C19—H19	120.1
F6'—C5—F5'	106.86 (14)	C19—C20—C21	120.3 (3)
F4—C5—F5'	64.2 (3)	C19—C20—H20	119.9
F6—C5—F5'	127.3 (4)	C21—C20—H20	119.9
F6'—C5—F4'	106.77 (15)	C16—C21—C20	120.1 (3)
F4—C5—F4'	46.5 (3)	C16—C21—H21	119.9
F6—C5—F4'	66.4 (4)	C20—C21—H21	119.9
F5'—C5—F4'	106.66 (14)	F4'—F4—C5	66.82 (18)
F6'—C5—F5	69.7 (4)	F4'—F4—F5'	119.4 (3)
F4—C5—F5	106.75 (14)	C5—F4—F5'	57.93 (16)
F6—C5—F5	106.59 (14)	F5'—F5—C5	68.07 (17)
F4'—C5—F5	139.5 (4)	F5'—F5—F6'	114.9 (4)
F6'—C5—C4	109.4 (4)	C5—F5—F6'	55.1 (2)
F4—C5—C4	112.7 (3)	F6'—F6—C5	69.2 (2)
F6—C5—C4	113.5 (3)	F6'—F6—F4'	124.3 (2)
F5'—C5—C4	117.6 (3)	C5—F6—F4'	56.8 (2)
F4'—C5—C4	109.0 (3)	F4—F4'—C5	66.69 (17)
F5—C5—C4	110.1 (3)	F4—F4'—F6	115.9 (4)
F7—C6—F8	107.26 (13)	C5—F4'—F6	56.8 (2)
F7—C6—F9	106.60 (13)	F5—F5'—C5	68.22 (18)
F8—C6—F9	106.74 (13)	F5—F5'—F4	124.5 (2)
F7—C6—C7	112.20 (19)	C5—F5'—F4	57.91 (16)
F8—C6—C7	111.06 (18)	F6—F6'—C5	69.3 (2)
F9—C6—C7	112.64 (19)	F6—F6'—F5	118.1 (4)
O3—C7—C8	128.7 (2)	C5—F6'—F5	55.2 (2)
			
O4—Cu1—O1—C2	−124.4 (2)	O4—Cu1—C8—C9	−1.75 (16)
O5^i^—Cu1—O1—C2	74.2 (2)	O1—Cu1—C8—C9	−7.0 (2)
O2—Cu1—O1—C2	−23.3 (2)	O5^i^—Cu1—C8—C9	151.74 (16)
O5—Cu1—O1—C2	151.0 (2)	O2—Cu1—C8—C9	−100.53 (17)
O3—Cu1—O2—C4	−158.9 (2)	O5—Cu1—C8—C9	82.53 (17)
O4—Cu1—O2—C4	108.8 (2)	Cu1—O4—C9—C8	−4.4 (4)
O1—Cu1—O2—C4	19.5 (2)	Cu1—O4—C9—C10	176.38 (17)
O5^i^—Cu1—O2—C4	−66.9 (2)	C7—C8—C9—O4	1.0 (5)
O4—Cu1—O3—C7	1.8 (2)	Cu1—C8—C9—O4	2.5 (2)
O5^i^—Cu1—O3—C7	163.5 (2)	C7—C8—C9—C10	−179.8 (3)
O2—Cu1—O3—C7	−99.2 (2)	Cu1—C8—C9—C10	−178.3 (3)
O5—Cu1—O3—C7	86.5 (2)	C7—C8—C9—Cu1	−1.5 (3)
O3—Cu1—O4—C9	2.8 (2)	O3—Cu1—C9—O4	−177.1 (2)
O1—Cu1—O4—C9	178.4 (2)	O1—Cu1—C9—O4	−1.7 (2)
O5^i^—Cu1—O4—C9	−102.3 (3)	O5^i^—Cu1—C9—O4	131.5 (2)
O2—Cu1—O4—C9	91.0 (2)	O2—Cu1—C9—O4	−93.2 (2)
O5—Cu1—O4—C9	−90.5 (2)	O5—Cu1—C9—O4	87.5 (2)
O3—Cu1—O5—N1	−125.16 (16)	O3—Cu1—C9—C8	−0.53 (17)
O4—Cu1—O5—N1	−32.87 (16)	O4—Cu1—C9—C8	176.5 (3)
O1—Cu1—O5—N1	56.90 (16)	O1—Cu1—C9—C8	174.81 (16)
O5^i^—Cu1—O5—N1	143.3 (2)	O5^i^—Cu1—C9—C8	−52.0 (3)
O3—Cu1—O5—Cu1^i^	91.59 (9)	O2—Cu1—C9—C8	83.36 (18)
O4—Cu1—O5—Cu1^i^	−176.12 (9)	O5—Cu1—C9—C8	−95.98 (17)
O1—Cu1—O5—Cu1^i^	−86.35 (8)	O3—Cu1—C9—C10	176.8 (3)
O5^i^—Cu1—O5—Cu1^i^	0.0	O4—Cu1—C9—C10	−6.1 (3)
Cu1^i^—O5—N1—C11	79.3 (3)	O1—Cu1—C9—C10	−7.9 (3)
Cu1—O5—N1—C11	−56.3 (3)	O5^i^—Cu1—C9—C10	125.3 (3)
Cu1^i^—O5—N1—C15	−104.5 (2)	O2—Cu1—C9—C10	−99.3 (3)
Cu1—O5—N1—C15	119.9 (2)	O5—Cu1—C9—C10	81.4 (3)
Cu1—O5—N1—Cu1^i^	−135.6 (2)	O4—C9—C10—F10	59.0 (3)
Cu1—O1—C2—C3	18.9 (4)	C8—C9—C10—F10	−120.3 (3)
Cu1—O1—C2—C1	−162.19 (14)	Cu1—C9—C10—F10	62.7 (4)
F3—C1—C2—O1	116.4 (2)	O4—C9—C10—F11	−59.7 (3)
F1—C1—C2—O1	−3.3 (3)	C8—C9—C10—F11	121.0 (3)
F2—C1—C2—O1	−124.8 (2)	Cu1—C9—C10—F11	−55.9 (4)
F3—C1—C2—C3	−64.5 (3)	O4—C9—C10—F12	179.4 (2)
F1—C1—C2—C3	175.8 (2)	C8—C9—C10—F12	0.1 (4)
F2—C1—C2—C3	54.3 (3)	Cu1—C9—C10—F12	−176.8 (2)
F3—C1—C2—Cu1	98.8 (3)	O5—N1—C11—C12	175.6 (2)
F1—C1—C2—Cu1	−20.9 (3)	C15—N1—C11—C12	−0.5 (4)
F2—C1—C2—Cu1	−142.3 (3)	Cu1^i^—N1—C11—C12	−148.8 (2)
O3—Cu1—C2—O1	179.0 (2)	N1—C11—C12—C13	−0.8 (4)
O4—Cu1—C2—O1	57.4 (2)	C11—C12—C13—C14	1.9 (4)
O5^i^—Cu1—C2—O1	−103.6 (2)	C11—C12—C13—C16	−177.1 (2)
O2—Cu1—C2—O1	154.8 (2)	C12—C13—C14—C15	−1.7 (4)
O5—Cu1—C2—O1	−30.8 (2)	C16—C13—C14—C15	177.3 (2)
O3—Cu1—C2—C3	13.4 (3)	C11—N1—C15—C14	0.7 (4)
O4—Cu1—C2—C3	−108.30 (17)	O5—N1—C15—C14	−175.5 (2)
O1—Cu1—C2—C3	−165.7 (3)	Cu1^i^—N1—C15—C14	145.5 (2)
O5^i^—Cu1—C2—C3	90.73 (17)	C13—C14—C15—N1	0.5 (4)
O2—Cu1—C2—C3	−10.82 (16)	C12—C13—C16—C21	142.6 (3)
O5—Cu1—C2—C3	163.51 (16)	C14—C13—C16—C21	−36.3 (4)
O3—Cu1—C2—C1	−151.5 (2)	C12—C13—C16—C17	−37.6 (4)
O4—Cu1—C2—C1	86.9 (3)	C14—C13—C16—C17	143.5 (3)
O1—Cu1—C2—C1	29.5 (2)	C21—C16—C17—C18	−0.2 (4)
O5^i^—Cu1—C2—C1	−74.1 (3)	C13—C16—C17—C18	179.9 (3)
O2—Cu1—C2—C1	−175.7 (3)	C16—C17—C18—C19	0.1 (5)
O5—Cu1—C2—C1	−1.3 (3)	C17—C18—C19—C20	0.0 (5)
O1—C2—C3—C4	1.0 (5)	C18—C19—C20—C21	0.0 (5)
C1—C2—C3—C4	−177.9 (2)	C17—C16—C21—C20	0.3 (4)
Cu1—C2—C3—C4	11.5 (3)	C13—C16—C21—C20	−179.9 (3)
Cu1—O2—C4—C3	−9.7 (4)	C19—C20—C21—C16	−0.2 (5)
Cu1—O2—C4—C5	171.25 (17)	F6'—C5—F4—F4'	66.1 (6)
C2—C3—C4—O2	−4.6 (5)	F6—C5—F4—F4'	30.2 (6)
C2—C3—C4—C5	174.4 (2)	F5'—C5—F4—F4'	154.1 (6)
C2—C3—C4—Cu1	−10.7 (3)	F5—C5—F4—F4'	144.0 (6)
O3—Cu1—C4—O2	22.0 (2)	C4—C5—F4—F4'	−95.1 (6)
O4—Cu1—C4—O2	−76.0 (2)	F6'—C5—F4—F5'	−88.0 (4)
O1—Cu1—C4—O2	−159.0 (2)	F6—C5—F4—F5'	−123.8 (5)
O5^i^—Cu1—C4—O2	114.2 (2)	F4'—C5—F4—F5'	−154.1 (6)
O5—Cu1—C4—O2	168.04 (19)	F5—C5—F4—F5'	−10.1 (5)
O3—Cu1—C4—C3	−165.59 (16)	C4—C5—F4—F5'	110.8 (4)
O4—Cu1—C4—C3	96.36 (16)	F6'—C5—F5—F5'	146.6 (6)
O1—Cu1—C4—C3	13.38 (16)	F4—C5—F5—F5'	13.2 (6)
O5^i^—Cu1—C4—C3	−73.38 (16)	F6—C5—F5—F5'	127.1 (6)
O2—Cu1—C4—C3	172.4 (3)	F4'—C5—F5—F5'	54.2 (9)
O5—Cu1—C4—C3	−19.6 (3)	C4—C5—F5—F5'	−109.4 (6)
O3—Cu1—C4—C5	3.6 (4)	F4—C5—F5—F6'	−133.4 (5)
O4—Cu1—C4—C5	−94.5 (4)	F6—C5—F5—F6'	−19.4 (5)
O1—Cu1—C4—C5	−177.4 (4)	F5'—C5—F5—F6'	−146.6 (6)
O5^i^—Cu1—C4—C5	95.8 (4)	F4'—C5—F5—F6'	−92.3 (5)
O2—Cu1—C4—C5	−18.4 (3)	C4—C5—F5—F6'	104.0 (4)
O5—Cu1—C4—C5	149.6 (3)	F4—C5—F6—F6'	141.9 (6)
O2—C4—C5—F6'	−55.4 (6)	F5'—C5—F6—F6'	71.9 (6)
C3—C4—C5—F6'	125.4 (6)	F4'—C5—F6—F6'	165.4 (7)
Cu1—C4—C5—F6'	−42.6 (7)	F5—C5—F6—F6'	28.1 (6)
O2—C4—C5—F4	110.8 (5)	C4—C5—F6—F6'	−93.2 (7)
C3—C4—C5—F4	−68.4 (5)	F6'—C5—F6—F4'	−165.4 (7)
Cu1—C4—C5—F4	123.6 (5)	F4—C5—F6—F4'	−23.5 (4)
O2—C4—C5—F6	−10.8 (4)	F5'—C5—F6—F4'	−93.5 (4)
C3—C4—C5—F6	170.0 (4)	F5—C5—F6—F4'	−137.3 (4)
Cu1—C4—C5—F6	2.0 (6)	C4—C5—F6—F4'	101.3 (3)
O2—C4—C5—F5'	−177.5 (5)	F5'—F4—F4'—C5	25.2 (5)
C3—C4—C5—F5'	3.3 (5)	C5—F4—F4'—F6	−29.3 (4)
Cu1—C4—C5—F5'	−164.7 (5)	F5'—F4—F4'—F6	−4.1 (8)
O2—C4—C5—F4'	61.0 (5)	F6'—C5—F4'—F4	−138.2 (6)
C3—C4—C5—F4'	−118.2 (5)	F6—C5—F4'—F4	−148.3 (6)
Cu1—C4—C5—F4'	73.8 (6)	F5'—C5—F4'—F4	−24.3 (6)
O2—C4—C5—F5	−130.2 (4)	F5—C5—F4'—F4	−60.1 (8)
C3—C4—C5—F5	50.6 (4)	C4—C5—F4'—F4	103.7 (5)
Cu1—C4—C5—F5	−117.3 (5)	F6'—C5—F4'—F6	10.0 (5)
Cu1—O3—C7—C8	−5.6 (4)	F4—C5—F4'—F6	148.3 (6)
Cu1—O3—C7—C6	174.94 (14)	F5'—C5—F4'—F6	124.0 (5)
F7—C6—C7—O3	2.6 (3)	F5—C5—F4'—F6	88.2 (5)
F8—C6—C7—O3	−117.5 (2)	C4—C5—F4'—F6	−108.1 (3)
F9—C6—C7—O3	122.9 (2)	F6'—F6—F4'—F4	15.9 (10)
F7—C6—C7—C8	−177.0 (2)	C5—F6—F4'—F4	32.5 (5)
F8—C6—C7—C8	63.0 (3)	F6'—F6—F4'—C5	−16.5 (7)
F9—C6—C7—C8	−56.7 (3)	F6'—F5—F5'—C5	29.9 (4)
F7—C6—C7—Cu1	8.2 (4)	C5—F5—F5'—F4	−14.5 (6)
F8—C6—C7—Cu1	−111.8 (3)	F6'—F5—F5'—F4	15.4 (9)
F9—C6—C7—Cu1	128.5 (3)	F6'—C5—F5'—F5	−32.7 (7)
O4—Cu1—C7—O3	−178.1 (2)	F4—C5—F5'—F5	−165.9 (7)
O1—Cu1—C7—O3	166.4 (2)	F6—C5—F5'—F5	−73.9 (5)
O5^i^—Cu1—C7—O3	−17.9 (2)	F4'—C5—F5'—F5	−146.6 (6)
O2—Cu1—C7—O3	80.6 (2)	C4—C5—F5'—F5	90.8 (6)
O5—Cu1—C7—O3	−94.6 (2)	F6'—C5—F5'—F4	133.3 (5)
O3—Cu1—C7—C8	175.6 (3)	F6—C5—F5'—F4	92.1 (3)
O4—Cu1—C7—C8	−2.47 (17)	F4'—C5—F5'—F4	19.3 (5)
O1—Cu1—C7—C8	−17.9 (3)	F5—C5—F5'—F4	165.9 (7)
O5^i^—Cu1—C7—C8	157.75 (17)	C4—C5—F5'—F4	−103.3 (3)
O2—Cu1—C7—C8	−103.75 (18)	F4'—F4—F5'—F5	−11.6 (11)
O5—Cu1—C7—C8	81.09 (18)	C5—F4—F5'—F5	15.9 (7)
O3—Cu1—C7—C6	−9.1 (3)	F4'—F4—F5'—C5	−27.5 (6)
O4—Cu1—C7—C6	172.8 (3)	F4'—F6—F6'—C5	14.8 (7)
O1—Cu1—C7—C6	157.4 (3)	C5—F6—F6'—F5	−26.6 (5)
O5^i^—Cu1—C7—C6	−27.0 (3)	F4'—F6—F6'—F5	−11.8 (10)
O2—Cu1—C7—C6	71.5 (3)	F4—C5—F6'—F6	−57.8 (7)
O5—Cu1—C7—C6	−103.6 (3)	F5'—C5—F6'—F6	−127.8 (6)
O3—C7—C8—C9	4.6 (5)	F4'—C5—F6'—F6	−13.9 (6)
C6—C7—C8—C9	−175.9 (2)	F5—C5—F6'—F6	−151.2 (6)
Cu1—C7—C8—C9	1.5 (3)	C4—C5—F6'—F6	103.9 (6)
O3—C7—C8—Cu1	3.1 (2)	F4—C5—F6'—F5	93.5 (5)
C6—C7—C8—Cu1	−177.4 (2)	F6—C5—F6'—F5	151.2 (6)
O3—Cu1—C8—C7	−2.18 (16)	F5'—C5—F6'—F5	23.4 (4)
O4—Cu1—C8—C7	176.8 (2)	F4'—C5—F6'—F5	137.3 (4)
O1—Cu1—C8—C7	171.52 (16)	C4—C5—F6'—F5	−104.9 (3)
O5^i^—Cu1—C8—C7	−29.7 (2)	F5'—F5—F6'—F6	−3.6 (8)
O2—Cu1—C8—C7	77.99 (18)	C5—F5—F6'—F6	30.7 (6)
O5—Cu1—C8—C7	−98.95 (18)	F5'—F5—F6'—C5	−34.3 (5)
O3—Cu1—C8—C9	179.3 (2)		

Symmetry codes: (i) −*x*+2, −*y*, −*z*.
